# Impact of Tranexamic Acid on Traumatic Hemorrhage Outcomes in Emergency Medicine: A Systematic Review and Meta-Analysis

**DOI:** 10.7759/cureus.93362

**Published:** 2025-09-27

**Authors:** Hany A Zaki, Waseem Ahmad, Mujeeb Ur Rehman, Hussam Elmelliti, Ali Elkandow, Mohammed Gafar Abdelrahim

**Affiliations:** 1 Emergency Department, Qatar University - College of Medicine, Doha, QAT; 2 Emergency Department, Hamad Medical Corporation, Doha, QAT; 3 Emergency Department, Hamad Medical Corporation, Al Khor, QAT

**Keywords:** acid tranexamic, antifibrinolytic agents, multiple trauma, trauma, traumatic injuries

## Abstract

Hemorrhage is the predominant cause of death among trauma patients. Therefore, pharmacological treatments such as tranexamic acid (TXA) have been explored to supplement the conventional measures of controlling bleeding in this population. TXA has been found beneficial among patients undergoing elective surgery. However, its use to mitigate bleeding among trauma patients in different settings remains controversial. Therefore, we carried out this study to examine the efficacy of prehospital and in-hospital TXA among trauma patients in military and civilian settings.

PubMed, MEDLINE, Cochrane Center Register of Controlled Trials (CENTRAL), ProQuest, and Google Scholar databases were searched for observational and randomized controlled trials (RCTs) related to our topic. Two authors independently abstracted the data required for review and analysis and recorded them in a standardized table. When possible, meta-analysis was performed using the Review Manager software (RevMan 5.4.1, The Cochrane Collaboration, London, UK).

Of the 5383 articles obtained from the database search, only 22 met our inclusion criteria. Data pooled from five of these studies showed that prehospital TXA had a non-significant decrease in mortality (OR: 0.91; 95% CI: 0.71 - 1.17; p = 0.45; I^2^ = 0) and no significant increase in thromboembolic events (OR: 2.96; 95% CI: 0.66 - 13.20; p = 0.16). However, prehospital TXA was associated with a significantly lower amount of blood transfusion (MD: -1.36; 95% CI: -5.36 to -2.16; p <0.00001). The pooled analysis also showed that TXA administered in hospitals is associated with significantly lower all-cause mortality (OR: 0.86; 95% CI: 0.76 - 0.98; p = 0.02) and mortality due to hemorrhage (OR: 0.84; 95% CI: 0.74 - 0.95; p = 0.005). However, in military settings, it was associated with increased incidences of thromboembolic events (OR: 3.22; 95% CI: 1.37 - 7.57; p = 0.007). Additionally, the pooled analysis has shown that a second dose of TXA has no mortality benefit (OR: 0.96; 95% CI: 0.21 - 4.41; p = 0.96 and OR: 1.51; 95% CI: 0.62 - 3.66; p = 0.36, for 24-hour and 28-day mortality, respectively) even though it does not increase incidences of thromboembolic complications (OR: 2.28; 95% CI: 0.48 - 10.81; p = 0.30). On the other hand, pooled analysis of TXA effects in patients undergoing massive transfusion has shown a trend of significantly lower mortality. However, it was associated with an increased risk of thromboembolic complications.

Prehospital TXA appears to have a non-significant reduction in mortality. However, we believe that additional RCTs might result in a significant difference. On the other hand, TXA administered in hospitals appears to reduce mortality in trauma patients with hemorrhage. However, we could not determine who was more likely to benefit from the treatment between civilians and the military. Moreover, we found that a second dose of TXA has no benefit among trauma patients; therefore, we suggest that clinicians withhold subsequent doses unless hyperfibrinolysis is observed. Lastly, TXA is beneficial for patients undergoing massive transfusion; therefore, we suggest that TXA be administered as early as possible in these patients.

## Introduction and background

Trauma accounts for close to 6 million fatalities worldwide every year, which is higher than mortality from all communicable diseases (i.e., HIV/AIDs, Tuberculosis, and COVID-19) combined [1]. Research has indicated that approximately a third of these deaths are attributed to hemorrhage [2,3]. Therefore, interventions that mitigate trauma-related bleeding are critical for the survival of these individuals. The conventional measures used for regulating hemorrhage in trauma patients include the administration of fresh frozen plasma, packed red blood cells, platelets, and cryoprecipitate [4,5]. However, these treatments involve transfusions and are linked with different complications, including transfusion reactions, metabolic complications, infectious complications, immunosuppression, transfusion-related acute lung injury, and multiple organ dysfunction [6]. As a result, the hunt for pharmacological agents, such as tranexamic acid (TXA), has escalated to improve on the traditional bleeding control therapies in traumatic settings.

TXA is a synthetic derivative of the amino acid lysine that functions as an antifibrinolytic drug to prevent traumatic exsanguination. It was originally used in the late 1960s to decrease menstrual bleeding and hemorrhage resulting from tooth extraction in hemophiliacs. However, its usage was eventually broadened to the treatment of hyperfibrinolysis, where it was proven to minimize blood loss and the requirement for blood transfusion [7]. Recently, studies have revealed that TXA is also useful for blood control following elective surgery. This conclusion is validated by a previous meta-analysis where TXA treatment decreased the requirement for transfusion by a third (relative risk (RR): 0.61, 95% CI: 0.54 - 0.70) without any notable reduction in mortality rate (RR: 0.61; 0.32 - 1.12)[8]. Moreover, the most recent major studies have revealed that TXA has a potential advantage in treating traumatic bleeding in civilian and military settings [9,10]. However, the evidence on trauma patients likely to benefit from the TXA therapy is contested. Therefore, the present meta-analysis was undertaken to investigate whether prehospital or in-hospital TXA could potentially benefit adult trauma patients in military and civilian settings. In addition, the research explored the advantages of a second dose of TXA and the benefits among patients undergoing massive transfusion, as well as the potential safety concerns associated with TXA use.

Therefore, the present systematic review and meta-analysis were undertaken to investigate the role of tranexamic acid in traumatic hemorrhage. The objectives were to evaluate the efficacy and safety of TXA administered in prehospital compared to in-hospital settings, to determine whether a second dose provides additional benefit, and to assess outcomes among patients undergoing massive transfusion protocols. The expected outcomes were reductions in all-cause and hemorrhage-related mortality, decreased transfusion requirements, and a clearer understanding of the risk of thromboembolic complications across different clinical contexts.

## Review

Methodology

Inclusion Criteria

This review was structured using the PICO framework. The population included adult trauma patients aged 18 years or older presenting with hemorrhage in either civilian or military settings. The intervention of interest was TXA administration in prehospital or in-hospital settings, regardless of dose, regimen, or timing, including single or repeated doses. The comparison was standard trauma care or no TXA administration. The outcomes evaluated were all-cause mortality and mortality due to hemorrhage as the primary outcomes. In contrast, secondary outcomes included the need for blood transfusion, thromboembolic events such as deep vein thrombosis, pulmonary embolism, and other vascular occlusive complications, in addition to overall safety outcomes.

Exclusion Criteria

We excluded studies that involved non-traumatic hemorrhage (such as surgical, postpartum, or gastrointestinal bleeding), pediatric populations, or those published in languages other than English. Case reports, case series, study protocols, letters to the editor, and systematic reviews were also excluded from the analysis. 

Eligibility Criteria

Two reviewers independently assessed all identified articles for eligibility. Studies were included if they were published in English and conducted on adult human patients aged 18 years or older. They investigated the use of TXA in various doses across different clinical settings, such as prehospital, in-hospital, military, or civilian environments. Eligible studies were required to report at least one relevant outcome, including mortality, adverse events, or the need for blood transfusion, and to include a control group for comparison.

Articles were excluded if they involved patients with non-traumatic hemorrhage, such as surgical bleeding, postpartum hemorrhage, or gastrointestinal bleeding. In addition, studies were excluded if they were published as letters to the editor, systematic reviews or meta-analyses, study protocols, or case reports. Research that included patients with hyperfibrinolysis was also excluded from consideration.

Information Sources and Search Strategy

Two reviewers extensively searched for articles related to our topic on five electronic databases, including PubMed, MEDLINE, Cochrane Central Register of Controlled Trials (CENTRAL), ProQuest, and Google Scholar. The search strategy employed in these databases used the following MeSH terms: (“Tranexamic acid” OR “antifibrinolytic agents” OR “TXA” OR “tranexamic”) AND (“trauma” OR “traumatic injuries” OR “traumatic injuries” OR “severe trauma” OR “multiple trauma” OR “civilian trauma” OR “wounds and injuries”) AND (“hemorrhage” OR “bleeding” OR “massive transfusion” OR “massive hemorrhage” OR “uncontrolled bleeding”) AND (“mortality” OR “exsanguination” OR “deaths” OR “mortality” OR “thrombotic events” OR “adverse events” OR “complications” OR “vascular occlusive events” OR “Deep vein thrombosis” OR “pulmonary embolism” OR “blood products” OR “blood transfusion”). References of potentially relevant studies were also screened for additional studies that may have been missed in the database search. Moreover, the reviewers eliminated all exact or close duplicates and grey literature that would have undermined our scientific research.

Selection Process

After the database was completed, all records were imported to Covidence® Systematic Review Software (Covidence, Melbourne, Australia), where close or exact duplicates were eliminated. The two reviewers tasked with the literature search screened the articles based on the title, abstracts, and full texts. Any discrepancies during this process were resolved by consulting a third reviewer.

Data Collection and Data Items

Two independent reviewers abstracted data from all included studies and recorded them in duplicate tables. The data extracted were as follows: Author ID (year of publication and first author’s surname), study design, country, setting of TXA administration, TXA dose and route of administration, the window of administration, characteristics of the participants (i.e., sample size, mean/median age, and gender distribution), and measured outcomes. When discrepancies in the extracted data were recorded, the reviewers engaged in constructive discussions to resolve the discrepancies. If they could not agree, a third reviewer was consulted.

Quality Assessment

One experienced reviewer was tasked with assessing the quality of all included studies. Articles included in the present study were randomized controlled trials and observational studies; therefore, the quality appraisal was performed using two different tools. The Cochrane’s risk of bias tool within the Review Manager software (RevMan 5.4.1, The Cochrane Collaboration, London, UK) was used to appraise RCTs, while the Newcastle-Ottawa Scale was used to appraise observational studies [11]. The overall quality of evidence was also analyzed by converting the outcomes from the tools to AHRQ standards [12].

Outcome Measures and Definitions

The main outcome measured in the present study was mortality (including mortality due to bleeding). On the other hand, secondary outcomes included incidences of thromboembolic events and the amount/units of blood transfusion.

Massive transfusion protocol (MTP) was the transfusion of 10 or more units of packed red blood cells within the first 24 hours of injury. Additionally, mortality in our study was defined as all deaths before discharge from medical treatment facilities (i.e., in-hospital mortality).

Synthesis Methods

To synthesize data, we derived four PICO questions to summarize our outcomes. When applicable (i.e., outcomes from two studies or more), we carried out meta-analyses; otherwise, a narrative review of outcomes was provided. All the statistical analyses were performed using RevMan 5.4.1. In the analyses, the DerSimonian and Laird random-effects model was employed to counter all the anticipated heterogeneity and provide more conservative pooled effect sizes. For outcomes reported using continuous data, the pooled effect size was calculated in terms of mean difference (MD) and 95% confidence interval (CI). On the other hand, the effect size of dichotomous outcomes was calculated using the simple Odds Ratio (OR). In addition, the heterogeneity between study outcomes was calculated using the statistics, of which values above 50% were regarded as significant.

Results

Study Selection

**Figure 1:**
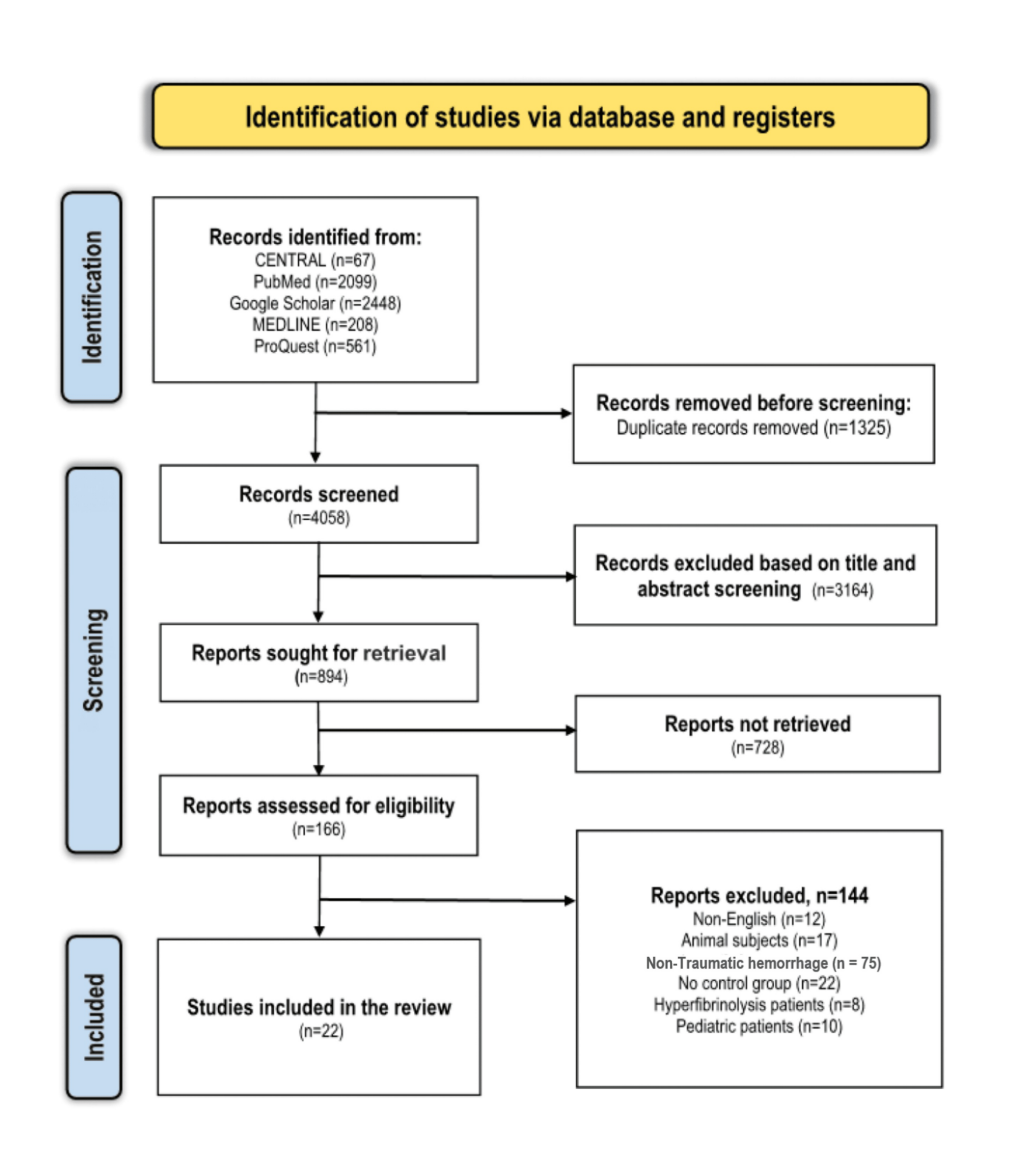
PRISMA flow diagram for study selection

Our search yielded 5383 articles with the predefined search terms. A duplicate analysis of these articles resulted in the exclusion of 1325 articles deemed as close or exact duplicates. Afterward, the screening criteria employed on the titles and abstracts of the remaining articles led to the exclusion of 3164 articles. Of the remaining 894 articles, we did not retrieve 728 because they were ongoing trials, conference abstracts, systematic reviews, letters to the editor, case reports, or case series. Consequently, only 22 articles met our criteria for inclusion, while the other articles were excluded for the following reasons: 17 included animal subjects, 10 were performed on pediatric patients, 12 were published in other languages, eight included hyperfibrinolysis patients, 22 had no control groups, and 75 included patients with non-traumatic hemorrhage. The full selection criteria are summarized using the PRISMA flow chart (Figure 1).

The characteristics of the included studies are detailed in Table 1.

**Table 1:** Characteristics of the included studies TXA: tranexamic acid; NR: not reported; RCT: randomized controlled trial

Author	Study design	Country	Participants’ characteristics			TXA dose and route of administration	Setting of TXA administration	Window of administration	Measured outcomes
			Sample (n)	M/F	Injury type (n)				
Shakur et al., 2010 [9]	RCT	40 countries worldwide	20207	16935/3271	Blunt (13665); penetrating (6552)	Loading dose of 1g over 10 minutes followed by IV infusion over 8 hours	Civilian	Within 8 hours of injury	Mortality, vascular occlusive events, units of transfused blood, and dependency.
Morrison et al., 2012 [10]	Retrospective observational study	Afghanistan	896	853/43	NR	Bolus of 1g TXA	Military	Within 24 hours of injury	Mortality and incidences of thrombotic events.
Shiraishi et al., 2017 [13]	Multicenter retrospective study	Japan	796	589/207	Blunt (794); penetrating (2)	IV TXA	Civilian	Within 3 hours of injury	Mortality, thromboembolic complications, and blood transfusion amounts.
Walker et al., 2020 [14]	Retrospective cohort study	United States	71	71/0	Penetrating TBI (36)	NR	military	NR	Mortality, thromboembolic incidences, blood transfusion amounts, and neurological outcomes
Neeki et al., 2017 [15]	Multicenter, prospective, observational cohort study	United States	253	207/46	Blunt (105); penetrating (148)	Loading dose of 1g TXA in 100ml of 0.9% saline over 10 minutes followed by IV infusion over 8 hours	Civilian	Within 3 hours of injury	Mortality, blood transfusion amount, and vascular occlusive events
Cole et al., 2015 [16]	Prospective cohort study	United Kingdom	385	160/225	Blunt (343); penetrating (42)	1g TXA infusion within 3 hours, followed by 1g when hemorrhage was detected	Civilian	Within 3 hours of injury	Mortality, organ failure, vascular occlusive events, and length of hospital stay
Boutonnet et al., 2018 [17]	Retrospective observational cohort study	France	797	142/650	Blunt (690); penetrating (79)	NR	Civilian	NR	Mortality
Lipsky et al., 2014 [18]	Observational study	Israel	40	35/5	Penetrating (22); blunt (18)	IV 1g TXA either by slow push (5-10 mins) or mixed with crystalloid for infusion	Military	Within 3 hours of injury	Mortality
Swendsen et al., 2013 [19]	Retrospective multicenter cohort study	United States	126	86/40	NR	1g bolus infusion over 10 minutes followed by a 1g infusion over 8 hours	Civilian	Within 3 hours of injury	Mortality and complications.
Adair et al., 2020 [20]	Retrospective observational study	United States	620	618/2	Blunt (53); penetrating (559); burn (8)	NR	Military	NR	Incidences of thrombotic events
Ng et al., 2019 [21]	Retrospective quantitative study	Canada	117	96/21	Blunt (123); penetrating (61)	Loading dose of 1g over 10 minutes followed by IV infusion over 8 hours	Civilian	Within 8 hours of injury	Blood transfusion amount, thromboembolic events, and mortality
Negahi et al., 2021 [22]	RCT	Iran	68	56/12	Blunt (68)	Ig TXA of intravascular infusion in 100 ccs of normal saline within 10 minutes followed by 1g every 12 hours up to 24 hours.	Civilian	Within 3 hours of injury	Blood transfusion amount, thromboembolic events, and mortality
Howard et al., 2017 [23]	Retrospective observational study	United States	3773	3629/144	NR	NR	Military	Within 3 hours of injury	Mortality and thrombotic events
El-Menyar et al., 2020 [24]	Retrospective study	Qatar	204	189/15	Blunt (186); penetrating (18)	Loading dose of 1g over 10 minutes followed by IV infusion over 8 hours	Civilian	Within 3 hours of injury	Blood transfusion amount, thromboembolic events, and mortality
Dixon et al., 2019 [25]	Single center retrospective study	United States	283	NR	Blunt (104)	Loading dose of 1g over 10 minutes followed by IV infusion over 8 hours	Civilian	Within 24 hours of injury	Mortality and thromboembolic events
Kovalev et al., 2021 [26]	Retrospective observational cohort study	United States	70	55/15	Blunt (70)	Loading dose of 1g over 10 minutes followed by IV infusion over 8 hours	Civilian	Within 3 hours of injury	Mortality and thromboembolic events
El-Menyar et al., 2022 [27]	Prospective, double-blind RCT	Qatar	220	211/9	Blunt (187); penetrating (26); both (7)	1g TXA in prehospital setting followed by 1g over 8 hours in hospital setting	Civilian		Mortality, thromboembolic complications, blood transfusions, and organ failure.
Johnston et al., 2018 [28]	Retrospective cohort study	Iraq and Afghanistan	455	443/12	Penetrating (407); blunt (48)	NR	Military	NR	Mortality, thromboembolic complications, and blood transfusions
Wafaisade et al., 2016 [29]	Cohort study	Germany	516	374/142	Blunt (473)	NR	Civilian	NR	Mortality and thromboembolic complications
Morrison et al., 2013 [30]	Retrospective observational study	Afghanistan	1332	1265/67	Penetrating (1273); blunt (59)	Loading dose of 1g bolus TXA followed by doses at clinicians’ discretion.	Military	NR	Mortality
Van Wessem et al., 2021 [31]	Prospective cohort study	Netherlands	422	298/124	Blunt (402); penetrating (20)	Loading dose of 1g bolus TXA followed by 1g repeated over 8 hours at the discretion of clinicians	Civilian	Within 3 hours of injury	Mortality and thromboembolic complications
Spinella et al., 2020 [32]	Double-blind, placebo-controlled RCT	United States	150	131/19	Penetrating (118); blunt (31)	2g or 4g of TXA in 40ml of normal saline over 10 minutes	Civilian	Within 2 hours of injury	Mortality and thromboembolic complications

Quality Appraisal Results

**Figure 2:**
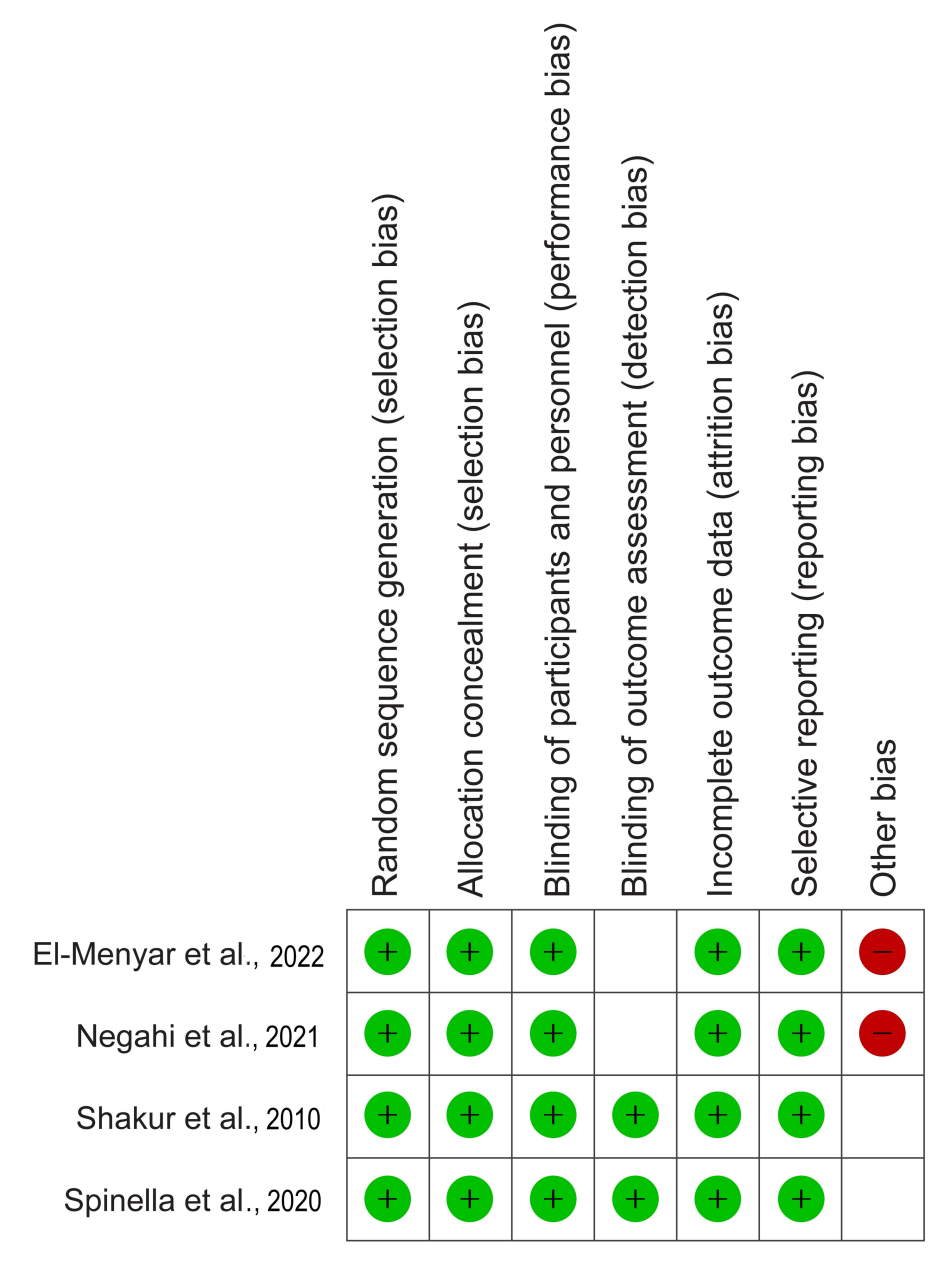
Risk of bias summary Shakur et al., 2010 [9], Negahi et al., 2021 [22], El-Menyar et al., 2022 [27], Spinella et al., 2020 [32].

The results of the risk of bias assessment and methodological quality, as assessed using the Newcastle-Ottawa scale, are summarized in Figure 2 and Table 2, respectively.

**Table 2:** Methodological quality using the Newcastle-Ottawa Scale

Study	Selection (/4)	Comparability (/2)	Outcome (/3)	Overall Quality
Shiraishi et al., 2017 [13]	2	2	2	Fair
Walker et al., 2020 [14]	3	2	2	Good
Neeki et al., 2017 [15]	2	2	2	Fair
Cole et al., 2015 [16]	2	2	2	Fair
Boutonnet et al., 2018 [17]	2	2	2	Fair
Lipsky et al., 2014 [18]	3	2	2	Good
Morrison et al., 2012 [10]	3	2	2	Good
Swendsen et al., 2013 [19]	3	2	2	Good
Adair et al., 2020 [20]	2	2	1	Fair
Ng et al., 2019 [21]	2	2	2	Fair
Howard et al., 2017 [23]	3	2	2	Good
Dixon et al., 2019 [25]	3	2	2	Good
Kovalev et al., 2021 [26]	2	2	2	Fair
El-Menyar et al., 2019 [34]	2	2	2	Fair
Johnston et al., 2018 [28]	2	2	2	Fair
Wafaisade et al., 2016 [29]	2	2	2	Fair
Morrison et al., 2013 [30]	3	2	2	Good
Van Wessem et al., 2021 [31]	2	2	2	Fair

The risk of bias assessment revealed that two RCTs [9,32] had a high quality, while the other 2 [22,27] had a moderate quality. Moreover, the quality appraisal using the Newcastle-Ottawa scale showed that most studies had fair quality due to a lack of blinding of participants and physicians, as well as insufficient information on exposure ascertainment. In contrast, only six studies [10,18,19, 23,25,30] had good methodological quality.

In patients with traumatic hemorrhage, is prehospital TXA effective and safe?

The European guidelines for the management of major bleeding and coagulopathy after trauma have recommended the early administration of TXA (within 3 hours of injury) to prevent bleeding and hyperfibrinolysis [33].

**Figure 3:**
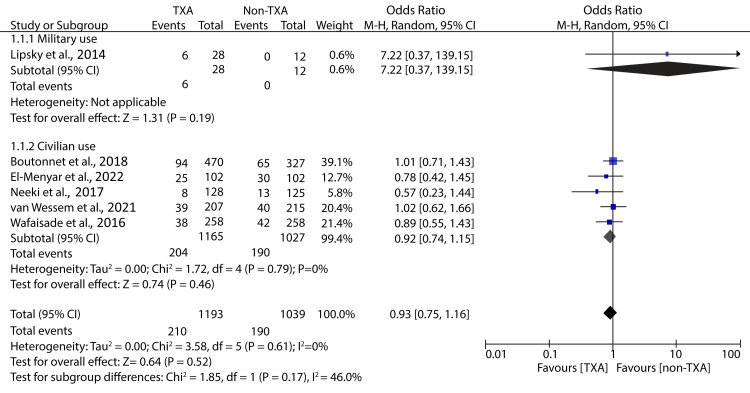
Forest plot showing the effect of prehospital TXA on All-cause mortality Neeki et al., 2017 [15], Boutonnet et al., 2018 [17], Lipsky et al., 2014 [18], El-Menyar et al., 2022 [27], Wafaisade et al., 2016 [29], Van Wessem et al., 2021 [31] TXA: tranexamic acid

Therefore, it is essential to investigate the effect of TXA administration at the point of injury or en route to the hospital. In our research, only five studies have discussed the impact of prehospital TXA in patients with traumatic hemorrhage. Data pooled from these studies has shown that prehospital TXA has no significant impact on all-cause mortality compared to no TXA administration (OR: 0.91; 95% CI: 0.71 - 1.17; p = 0.45; I² = 0%) (Figure 3). The subgroup analyses also revealed an insignificant difference in mortality between TXA and non-TXA groups in the military (OR: 7.22; 95% CI: 0.37 - 139.15; p = 0.19) and civilian settings (OR: 0.90; 95% CI: 0.70 - 1.15; p = 0.39) (Figure 3).

**Figure 4:**
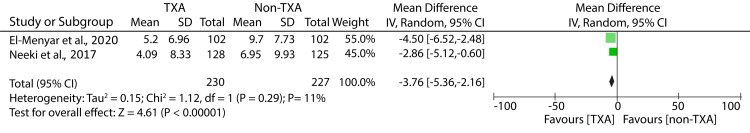
Forest plot showing the effect of prehospital TXA on Blood transfusion amounts Neeki et al., 2017 [15], El-Menyar et al., 2020 [24] TXA: tranexamic acid

On the other hand, our analysis found that prehospital TXA administration was associated with a significantly lower amount of blood transfusion products (MD: -1.36; 95% CI: -5.36 - -2.16; p < 0.00001) (Figure 4).

**Figure 5:**
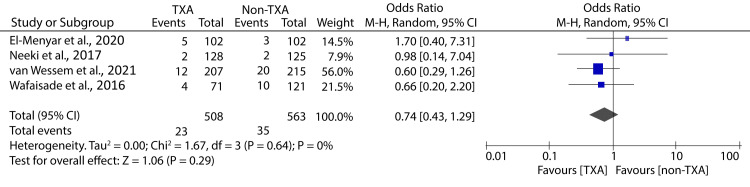
Forest plot showing the effect of TXA on all-cause mortality among patients undergoing massive transfusion El-Menyar et al., 2020 [24], Neeki et al., 2017 [15], van Wessem et al., 2021 [31], Wafaisade et al., 2016 [29] TXA: tranexamic acid

However, an insignificant difference in the incidences of thromboembolic complications between the TXA and non-TXA groups was recorded (OR: 2.96; 95% CI: 0.66 - 13.20; p = 0.16) (Figure 5).

Is TXA administration in the hospital effective and safe in patients with traumatic hemorrhage?

**Figure 6:**
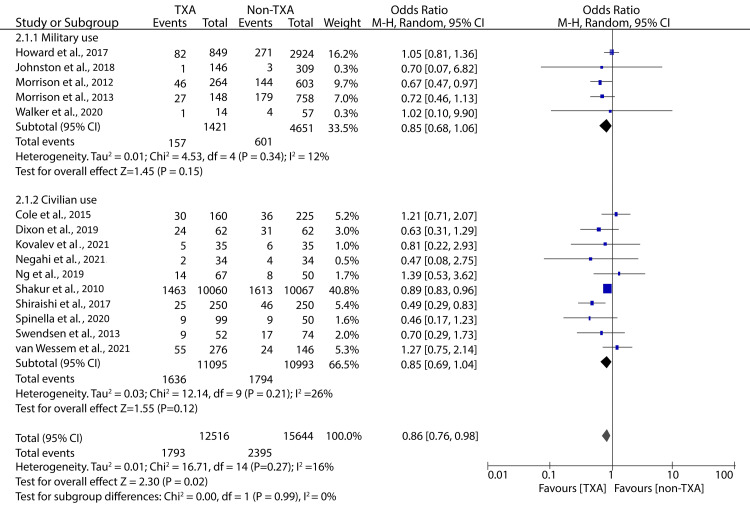
Forest plot showing the effect of in-hospital TXA on all-cause mortality Shakur et al., 2010 [9], Morrison et al., 2012 [10], Shiraishi et al., 2017 [13], Walker et al., 2020 [14], Cole et al., 2015 [16], Swendsen et al., 2013 [19], Ng et al., 2019 [21], Negahi et al., 2021 [22], Howard et al., 2017 [23], Dixon et al., 2019 [25], Kovalev et al., 2021 [26], Johnston et al., 2018 [28], Morrison et al., 2013 [30], Van Wessem et al., 2021 [31], Spinella et al., 2020 [32]. TXA: tranexamic acid

In-hospital TXA administration was reported in 16 studies included in our review article. The pooled analysis of outcomes from 14 of these studies showed that in-hospital TXA was associated with significantly lower mortality (OR: 0.86; 95% CI: 0.76 - 0.98; p = 0.02; n = 16%) (Figure 6). However, our subgroup analyses showed no significant difference in mortality between TXA and non-TXA groups in the military (OR: 0.85; 95% CI: 0.68 - 1.06; p = 0.15) and civilian settings (OR: 0.85; 95% CI: 0.69 - 1.04; p = 0.12) (Figure 6).

**Figure 7:**
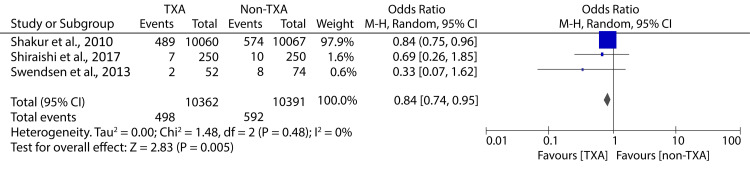
Forest plot showing the effect of in-hospital TXA on mortality due to hemorrhage Shakur et al., 2010 [9], Shiraishi et al., 2017 [13], Swendsen et al., 2013 [19] TXA: tranexamic acid

Further analyses also revealed that TXA administration in the hospital was associated with significantly reduced mortality due to hemorrhage (OR: 0.84; 95% CI: 0.74 - 0.95; p = 0.005) (Figure 7).

**Figure 8:**
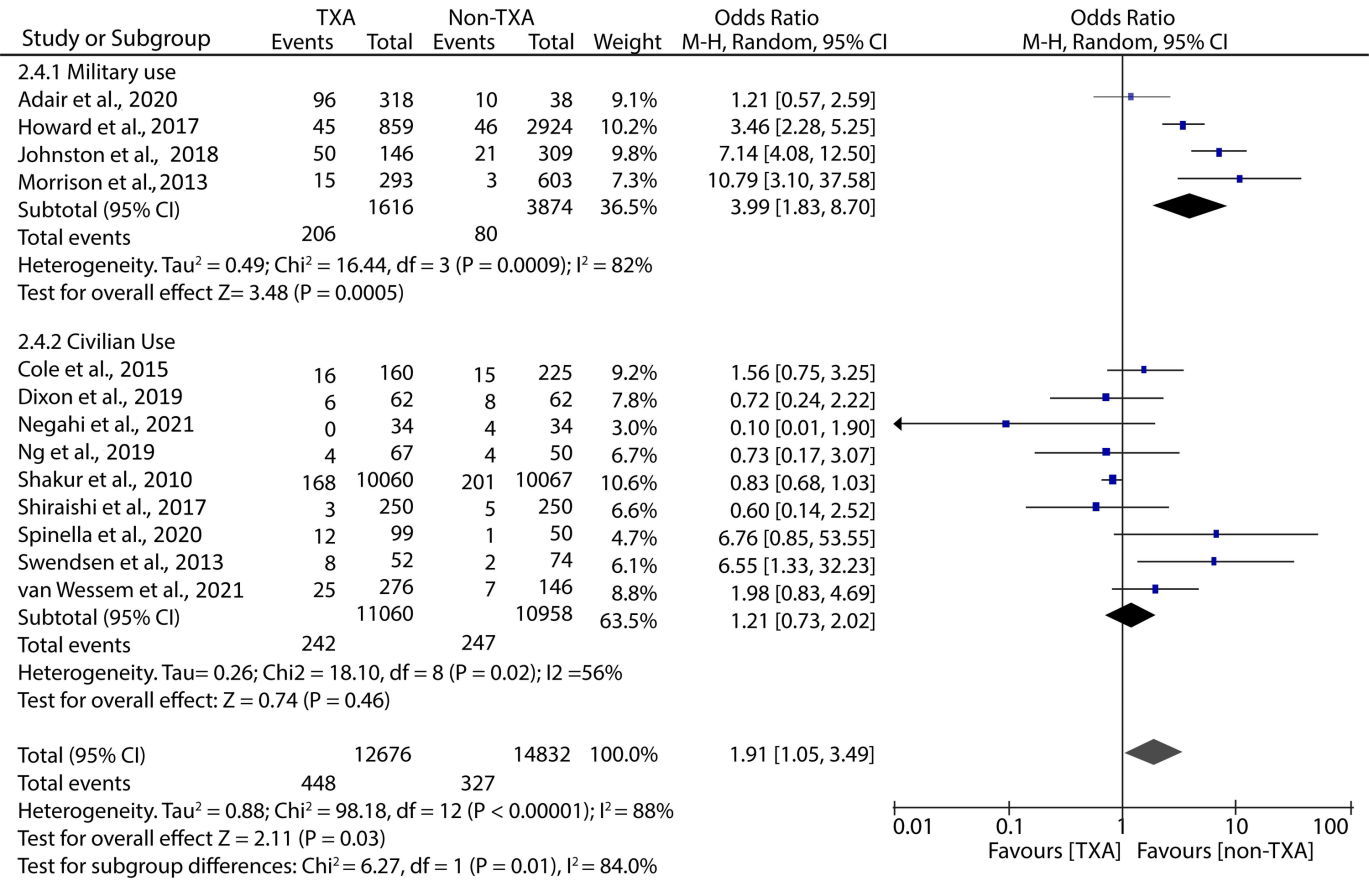
Forest plot showing the effect of in-hospital TXA on thromboembolic events Shakur et al., 2010 [9], Shiraishi et al., 2017 [13], Cole et al., 2015 [16], Swendsen et al., 2013 [19], Adair et al., 2019 [20], Ng et al., 2019 [21], Negahi et al., 2021 [22], Howard et al., 2017 [23], Dixon et al., 2019 [25], Johnston et al., 2018 [28], Morrison et al., 2013 [30], Van Wessem et al., 2021 [31], Spinella et al., 2020 [32] TXA: tranexamic acid

On the other hand, our subgroup analyses have shown that in a military setting, TXA administration in hospitals is associated with an increased incidence of thromboembolic complications (OR: 3.22; 95% CI: 1.37 - 7.57; p = 0.007). However, in a civilian setting, patients receiving TXA in hospitals have statistically similar incidences of thromboembolic complications as those not treated with TXA (OR: 1.21; 95% CI: 0.73 - 2.02; p = 0.46) (Figure 8).

Is a second dose of TXA administered in the hospital after the first dose was administered in a prehospital setting effective and safe?

**Figure 9:**
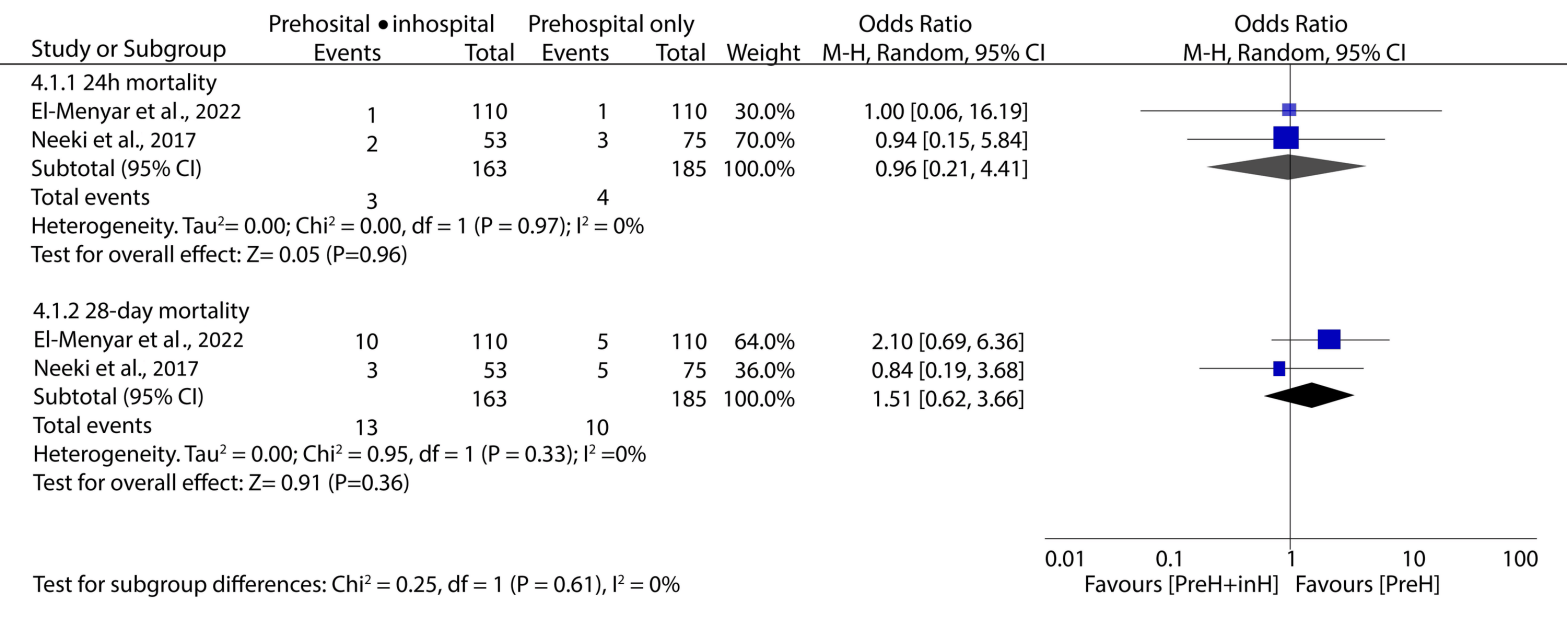
Forest plot showing the effect of a second dose of TXA on all-cause mortality Neeki et al., 2017 [15], El-Menyar et al., 2022 [27] TXA: tranexamic acid

Two studies included in the present study investigated whether a second dose of TXA administered in the hospital would improve the outcomes of traumatic hemorrhage patients. Data pooled from these studies has shown that a second dose of TXA administered in hospital has an insignificant effect on 24-hour and 28-day mortality compared to a single prehospital dose (OR: 0.96; 95% CI: 0.21 - 4.41; p = 0.96 and OR: 1.51; 95% CI: 0.62 - 3.66; p = 0.36, respectively) (Figure 9).

**Figure 10:**
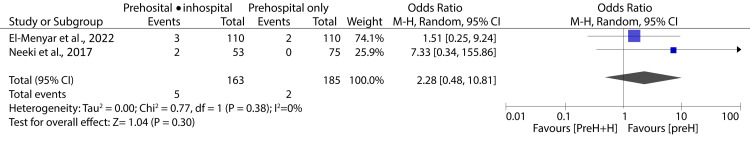
Forest plot showing the effect of a second dose of TXA on thromboembolic events Neeki et al., 2017 [15], El-Menyar et al., 2022 [27] TXA: tranexamic acid

Similarly, our pooled analysis did not record any significant difference in the incidence of thromboembolic events between patients receiving a second dose of TXA in the hospital and those who received only a prehospital dose (OR: 2.28; 95% CI: 0.48 - 10.81; p = 0.30) (Figure 10).

In traumatic hemorrhage patients requiring massive transfusion protocol (MTP), is TXA administered at any time beneficial and safe?

**Figure 11:**
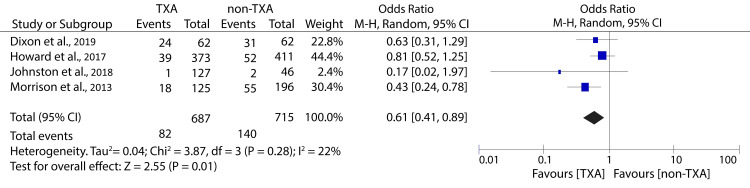
Forest plot showing the effect of TXA on all-cause mortality among patients undergoing massive transfusion Howard et al., 2017 [23], Dixon et al., 2019 [25], Johnston et al., 2018 [28], Morrison et al., 2013 [30]. TXA: tranexamic acid

In many trauma centers worldwide, massive transfusion has been identified as an indication of TXA administration. Therefore, one of our goals was to investigate the efficacy and safety of TXA administration in trauma patients undergoing MTP. Only four studies in the present review provided outcomes for patients undergoing MTP. The data pooled from these studies showed that TXA administration significantly reduced all-cause mortality (OR: 0.61; 95% CI: 0.41 - 0.89; p = 0.01) (Figure 11).

**Figure 12:**
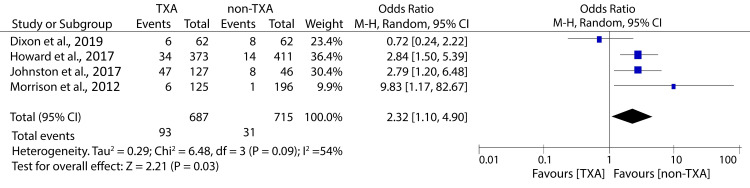
Forest plot showing the effect of TXA on thromboembolic events among patients undergoing massive transfusion Howard et al., 2017 [23], Dixon et al., 2019 [25], Johnston et al., 2018 [28], Morrison et al., 2012 [10] TXA: tranexamic acid

However, it was also associated with an increased incidence of thromboembolic complications (OR: 2.23; 95% CI: 1.10 - 4.90; p = 0.03) (Figure 12).

Discussion

This meta-analysis was primarily carried out to explore the therapeutic impact of TXA in patients suffering from traumatic bleeding. The statistical assessments indicated non-significant variation in mortality and thromboembolic complications between individuals treated with prehospital TXA and those who did not. However, we found that prehospital TXA administration has the potential to reduce the units of transfused blood. In contrast, our meta-analysis has demonstrated a substantial reduction in all-cause mortality and death owing to loss of blood for patients treated with TXA in hospitals. In the subgroup of patients receiving massive transfusion protocols (MTP), TXA was associated with a marked reduction in mortality but also with an increased incidence of thromboembolic events, which may be influenced by transfusion-related factors or trauma-induced coagulopathy, including disseminated intravascular coagulation (DIC).

Our findings on the effect of prehospital TXA on mortality and incidences of thromboembolic events are complemented by a previous systematic review of two studies with a pooled sample of 769 traumatic hemorrhage patients [34]. According to the pooled analysis in that study, the death rate after 30 days and thromboembolic incidents did not vary significantly between the populations receiving TXA and the control intervention (OR: 0.86; 95% CI: 0.559 - 1.32 and OR: 0.74; 95% CI: 0.267 - 2.066, respectively). Our meta-analysis found no statistically significant reduction in all-cause mortality with prehospital TXA administration; however, a numerical trend toward lower mortality was observed (17.6% vs. 18.5%). While not statistically significant, this finding suggests a potential clinical benefit. It highlights the need for further well-designed randomized trials to clarify the role of TXA in prehospital trauma care. Therefore, this accumulated evidence suggests that prehospital TXA might be effective and safe for trauma patients in civilian and military settings. However, there is a need for more randomized trials to establish its efficacy on mortality outcomes. As a result, we believe that the ongoing clinical trial on the efficacy of prehospital TXA will provide adequate power to obtain statistical significance in subsequent research [35].

Furthermore, we found that trauma patients treated with prehospital TXA received significantly fewer blood transfusions. This significant reduction can be explained by the fact that the swift TXA administration in prehospital settings provided ample time for the therapeutic effects of TXA to manifest. Therefore, prehospital TXA is beneficial for traumatic hemorrhage patients. Contrary to this finding, a recent randomized trial demonstrated an insignificant difference in the total amount of blood components transfused during the first 24 hours between patients receiving TXA and those who did not (p = 0.97) [36]. The insignificant difference in this trial can be attributed to the inclusion of patients at risk of hemorrhage only. Furthermore, the results of that trial do not establish causation, as it was statistically underpowered due to early termination following slow enrollment and financial constraints.

Our pooled analysis has also shown that in-hospital TXA resulted in a substantial reduction in all-cause mortality and mortality due to hemorrhage. This finding is disputed by a prior meta-analysis of 17 studies, which reported an insignificant variation in in-hospital mortality between TXA and non-TXA groups (p = 0.12) [37]. However, compared to our meta-analysis, their findings exhibited significant heterogeneity, implying their results were statistically underpowered. The study also combined the data for prehospital and in-hospital TXA; thus, it is possible that the insignificant difference was mostly driven by the outcomes from prehospital TXA admission, which has been shown to have an insignificant effect on mortality in our study. Considering the outcomes accumulated in our analysis, it is evident that in-hospital TXA is beneficial for treating traumatic hemorrhage. However, the subgroup analyses of patients in civilian and military settings have demonstrated statistically insignificant differences in mortality between TXA and non-TXA groups, indicating the need for more high-quality RCTs to establish which set of traumatic patients is more likely to benefit from the TXA administration.

In addition to reduced mortality, TXA administered in the hospital did not increase the risk of thromboembolic incidents. Therefore, our results indicate that in-hospital TXA is a reliable and safe therapeutic option for bleeding trauma patients. In the military setting, TXA administration was associated with an increased risk of thromboembolic events. This effect may be influenced by factors such as the mechanism of injury (projectile versus secondary blast injuries with shrapnel), anatomical wound location, and overall burden or number of wounds, all of which can alter the coagulation profile and risk of thrombosis. However, as these variables were not consistently reported in the included studies, we were unable to explore their impact in subgroup analyses. This finding is not surprising as TXA is provided with the intention to enhance clotting and minimize blood loss; thus, the risk for thromboembolic incidents is elevated. Additionally, most of the patients in military settings are severely injured; thus, the risk of developing complications in this population is elevated. Despite these potential safety concerns of TXA in military settings, the potential mortality benefit should not be traded off based on our results. This is because our outcomes have a significant heterogeneity (I2 = 82%); thus, they are statistically underpowered, and TXA may have proven safe to use in some military trauma patients. In addition, the large randomized trial CRASH-2 and MATTERs I and II studies have shown that the number needed to treat to generate a nonfatal thrombotic event (i.e., PE) is approximately 24, and the number needed to treat for a PE death was approximately 1887 [9,10]. Therefore, these estimates suggest that the potential mortality benefit of TXA might outweigh the potential risk for thromboembolic events.

Although we evaluated thromboembolic events as the only complications, it is crucial to emphasize the influence of TXA on organ failure, which has poor outcomes in trauma patients [38,39]. Cole and colleagues observed that TXA treatment coincided with lower occurrences of organ failure [16]. Moreover, they noted a statistically significant decrease in multiple organ failure after TXA was administered to patients in hemorrhagic shock. This decrease was observed even though the patients had coagulopathy at admission and increased blood transfusion, which are known to increase the risk of developing multiple organ failure [38-40]. On the other hand, the CRASH-2 trial found an insignificant difference in deaths due to multiple organ failure between the TXA and control groups [9]. Therefore, the evidence from these studies points to the safe treatment of trauma patients using TXA. However, this finding should be interpreted in light of the following limitations. The study by Cole was conducted in a single center, which may limit the generalizability of its outcomes [16].

Furthermore, the trauma center level (e.g., Level I versus lower-level facility) was not specified in detail, and this factor could influence outcomes given differences in resource availability, specialist coverage, and trauma system maturity. As this information was not consistently reported across studies, we were unable to stratify outcomes by trauma center designation. Secondly, the study had a small sample size bias. Finally, the CRASH-2 trial may have been statistically underpowered due to early termination [9].

The outcomes of the present review suggest that a second dose of TXA, whether liberal, protocolized, or optional, may not provide additional mortality benefit in traumatic hemorrhage patients. However, it is essential to note that the time to transfer and delays in access to definitive trauma care could influence redosing decisions in practice. In prolonged transfer scenarios, particularly in military or austere environments, clinicians may administer a second dose to maintain antifibrinolytic activity until surgical control of bleeding is achieved. As this variable was not consistently reported in the included studies, we could not assess its impact on outcomes. However, we noticed identical rates of thromboembolic events across individuals getting a second dosage of TXA in the hospital and those receiving just a prehospital dose, which indicates that the second dose of TXA has no safety concerns. Moreover, El-Menyar and colleagues reported an insignificant difference in blood transfusion needs between the in-hospital and prehospital TXA groups (p = 0.76) [27]. In contrast, Neeki et al. (2017) found that patients receiving a second dose of TXA required more units of blood products than those administered a prehospital dose [15]. This outcome is not unexpected given that patients received a second dose of TXA if they displayed signs and symptoms of hemorrhagic shock after the first dose. Considering this evidence, the traditional approach of delivering a second dosage of TXA is concerning and should be reconsidered.

Recently, TXA has been widely accepted as a treatment for patients undergoing massive transfusion due to its antifibrinolytic properties and ability to stabilize clots [41]. Therefore, it is important to evaluate its effectiveness in MTP. Our analysis has shown that TXA for patients undergoing MTP is associated with reduced mortality but increases the risk for thromboembolic complications. Despite the safety concerns, the potential mortality benefit reported in our study should not be ignored. This is because the heterogeneity in thromboembolic event outcomes was significant (I2 = 54%), suggesting that the statistical analyses were underpowered and more studies are required to establish the safety of TXA administration in this population fully. Contrary to our finding, one of the included studies found that TXA in trauma patients scheduled for massive transfusion had a non-significant reduction in mortality and did not increase the risk for thromboembolic complications [25]. However, the evidence in this study cannot be solely used to guide the clinical care of trauma patients undergoing massive transfusions due to various limitations. First, the study had low statistical power, meaning that the insignificant mortality benefit might be attributed to type II error. Besides, TXA protocolization was changed in 2015, meaning that the study included patients receiving pre- and post-protocolization TXA, which might explain their insignificant mortality benefit. Finally, the exact timing of TXA could not be determined and was only inferred from nursing chart records.

Although our scope did not encompass factors that influence the effects of TXA, evidence has shown that the timing of TXA administration is a key factor. The CRASH-2 trial suggested that early administration of TXA (within three hours after injury) might be more effective [9]. Although the statistical analyses on all-cause mortality did not support this finding, the investigators found that early administration of TXA was associated with reduced mortality due to bleeding. Furthermore, the Department of Defense Committee on Tactil Combat Casualty Care (CoTCCC) has recently recommended using TXA within three hours after injury [42]. On the other hand, a recent prospective trial of 70 trauma patients receiving prehospital TXA found a significantly lower production of fibrinogen fragments among patients receiving TXA [43]. This evidence implied that early TXA administration was associated with clot stabilization and reduced fibrinolytic activity. Therefore, contemporary evidence suggests that early TXA administration may have a mortality benefit without increasing the risk for thromboembolic events.

Limitations

When interpreting our results, a few limitations have to be taken into consideration. First, only studies published in English were eligible for review and analysis in our study. Therefore, data that would have been used to improve our statistical power were excluded because they were published in other languages. Second, significant heterogeneity was recorded in some analyses, especially those on thromboembolic events. However, the heterogeneity did not tamper with our findings. Moreover, the use of a random effect model was justified in these analyses. Third, our study included only adult trauma patients. Therefore, the outcomes cannot be extended to pediatric patients with traumatic hemorrhage. Fourth, most of the studies included in our study were observational, meaning that blinding the physician was impossible; therefore, their outcomes may have undermined the statistical power of our meta-analyses. Finally, due to missing information on the various causes of death among trauma patients receiving TXA in prehospital settings, we could not determine how it is associated with deaths resulting from hemorrhage.

We observed substantial variance in effect estimates between some studies, particularly Dixon et al. and Morrison et al., in the massive transfusion subgroup, which may reflect differences in dosing protocols, injury patterns, transfusion practices, or VTE detection methods. As these factors were not uniformly reported, this heterogeneity should be considered when interpreting our findings.

## Conclusions

The findings from our research indicate that prehospital administration of TXA does not provide a mortality benefit; however, it reduces the need for blood transfusions and appears safe as it does not increase thromboembolic risk. Although the difference in mortality was not statistically significant, patients treated with TXA showed a trend toward lower death rates, suggesting potential feasibility for trauma care in both civilian and military settings. Ongoing clinical trials may provide the statistical power needed to confirm these benefits. Moreover, in-hospital TXA use was associated with reduced mortality without an increase in thromboembolic events, supporting its role as an effective and safe treatment for traumatic hemorrhage. Notably, in military populations with more severe injuries, TXA was linked to higher thromboembolic complications, but the survival benefits should not be overlooked in this high-risk group.

Our analysis also demonstrated that a second dose of TXA offers no additional benefit in adult trauma patients and should be reserved only for those with confirmed hyperfibrinolysis. In contrast, trauma patients undergoing massive transfusion appear to benefit significantly from TXA, and early administration is strongly recommended in this subgroup. Collectively, these findings reinforce the importance of context-specific TXA use-early in massive transfusion protocols, cautiously in military settings, and with avoidance of unnecessary repeated dosing-to optimize patient outcomes.
